# Effects of housing conditions on health and gut microbiome of female cynomolgus monkeys and improvement of welfare by checking menstruation under socially housed condition

**DOI:** 10.1016/j.heliyon.2025.e41912

**Published:** 2025-01-12

**Authors:** Yunpeng Yang, Yong Lu, Changshan Gao, Yanhong Nie, Hongfei Wang, Yufei Huang, Haiyan Dong, Qiang Sun

**Affiliations:** a Jiangsu Co-innovation Center for Prevention and Control of Important Animal Infectious Diseases and Zoonoses, College of Veterinary Medicine, Yangzhou University, Yangzhou, 225009, China; b Institute of Comparative Medicine, Yangzhou University, Yangzhou, 225009, China; c Institute of Neuroscience, CAS Key Laboratory of Primate Neurobiology, State Key Laboratory of Neuroscience, CAS Center for Excellence in Brain Science and Intelligence Technology, Chinese Academy of Sciences, Shanghai, 200031, China; d Shanghai Center for Brain Science and Brain-Inspired Intelligence Technology, Shanghai, 201600, China

## Abstract

Laboratory non-human primates (NHPs) are commonly subjected to social deprivation in various scientific researches. However, the impact of social deprivation on gut microbiome remains largely unknown. We examined the health status and gut microbiota of female cynomolgus monkeys housed in isolation or social conditions and found that social deprivation brought adverse effects to monkeys by inhibiting their growth, remodeling the immune status, and decreasing the level of beneficial biochemical parameters. 16S rRNA gene sequencing revealed that the gut microbial composition and function differed between grouped and isolated monkeys. Specifically, grouping the single-caged young monkeys to socially housed condition could decrease the relative abundance of Firmicutes and increase the relative abundance of Bacteroidetes, while separating the socially housed middle-aged monkeys into single cages showed the opposite trend. Besides, training female monkeys to detect menstruation under socially-housed condition could increase their body weight change and adjusting their immune status, thus attenuating the adverse effects of separating them to single cages. Our results verified the significant role of grouping in mitigating adverse health and microbiota alterations caused by isolation in female cynomolgus monkeys and emphasized the importance of training NHPs to cooperate with experimental procedures under socially housed condition, which could not only improve the welfare of cynomolgus monkeys but also enhance the accuracy and reliability of scientific results.

## 1 Introduction

Environmental enrichment plays vital roles in maintaining the normal behavior and physiological state of laboratory animals, and ultimately enhances their well-being [[1], [2], [3]]. However, experimental animals face various environmental stressors during scientific research. Among these factors, the variation of housing conditions occurs frequently. In rodents, social deprivation can induce anxiety-like behaviors, leading to adverse effects on capacity for learning and memory [3,4]. In NHPs, these social animals are commonly subjected to a series of environmental stresses in scientific experiments. For example, to acquire oocytes for assisted reproductive technology (ART), female cynomolgus monkeys are commonly isolated into single cages for menstruation detection [5]. Besides, the NHPs should be housed in social deprivation conditions for a long time when they were subjected to the following experiments, e.g., evaluating the efficacy of drug therapy, investigating the food and water intake, and recording the corresponding parameters in neurobehavioral analysis, etc. Notably, the genetically modified NHPs that exhibited poor health state (e.g., BMAL1-deficient monkeys) [6] would also be housed singly. As previously reported, housing NHPs in individual cages (*i.e.*, single-cage housing) can affect their physical and physiological states, as well as inducing depression-like and autism-like behaviors [[7], [8], [9], [10]]. In addition, social isolation has been shown to increase the heart rate of female cynomolgus monkeys that fed with high cholesterol high fat diet, consequently potentiating atherosclerosis [11]. Moreover, housing conditions can also affect the immune status of NHPs [12]. Therefore, understanding the impact of housing conditions on the health status of NHPs has emerged as a critical issue for ensuring both their welfare, as well as their normal physiological status and behaviors.

Gut microbiota is complex microbial ecosystem that colonized in the gastrointestinal tract of animals. It can affect host fitness by modulating host behavior, nutritional status and immune function [[13], [14], [15]]. Previous studies have reported that social interactions exert significant influence on the composition of gut microbiota. For example, frequent social interactions in socially-housed primates can increase their gut microbial richness [16]. In rodents, social deprivation induces short-term alterations of gut microbiota in a sex-dependent manner [17]. Notably, gut microbiota is viewed as essential factor for the social development of mice [18,19]. In NHPs, although the gut microbiome has been reported to be affected by various factors (e.g., sex, aging, diet, habitat and seasonal factors) [[20], [21], [22], [23]], the influence of housing conditions on gut microbiome has been largely overlooked.

Here, we applied two strategies to investigate the impact of housing condition on the health state and gut microbiome of female cynomolgus monkeys. In the first strategy, the single-caged young monkeys were grouped into socially housed condition. In the second strategy, the socially housed middle-aged female cynomolgus monkeys were separated into single cages. By comparing the physical and physiological parameters of different monkey groups, we found that social deprivation could bring adverse effects to monkeys by inhibiting their growth, remodeling the immune status, and decreasing the level of beneficial biochemical parameters. 16S rRNA gene sequencing revealed that the gut microbial composition and function differed between grouped and isolated monkeys at specific time point. Besides, training female monkeys to detect menstruation under socially-housed condition attenuated the adverse effects of assigning them to single cages. In summary, our results not only deciphered the impact of housing conditions on the health state and gut microbiome of female cynomolgus monkeys, but also emphasized the importance of training NHPs to cooperate with experimental procedures under socially housed condition.

## 2 Results

### 2.1 Social deprivation impairs the health status of female cynomolgus monkeys

**Fig. 1:**
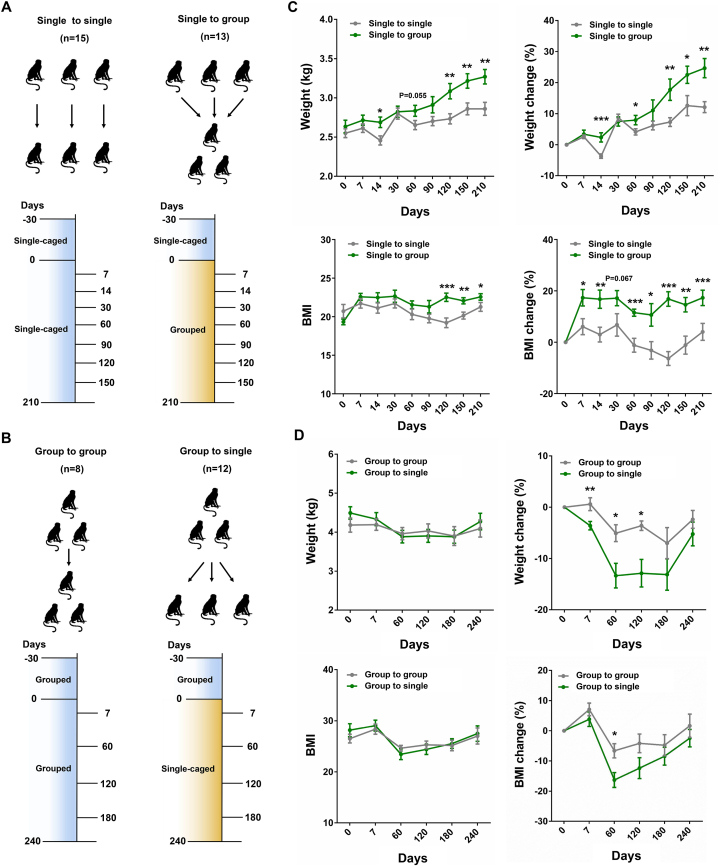
Examining the physical status of female cynomolgus monkeys housed in isolation and social conditions (A) Schematic diagram illustrating the strategies for grouping single-caged young monkeys to socially housed condition. Single to single, monkeys that housed in single cages before and during the experiment; Single to group, monkeys that housed in single cages before the experiment and then grouped to social housing condition during the experiment. The number of monkeys used in two monkey groups was listed in brackets. The monkeys used in this strategy were 2–3 years old. (B) Schematic diagram illustrating the strategies for separating grouped middle-aged monkeys into single cages. Group to group, monkeys that housed in social condition before and during the experiment; Group to single, monkeys that housed in social condition before the experiment and then divided into single cages during the experiment. The number of monkeys in two groups were listed in bracket. The monkeys used in this strategy were 10–15 years old. (C) The weight and BMI between the single-caged (single to single) and grouped (single to group) young monkeys. (D) The weight and BMI of middle-aged monkeys housed in social (group to group) and isolation (group to single) condition. Data are presented as mean ± SEM. The statistical significance between two monkey groups was analyzed using *t*-test (∗*p* < 0.05, ∗∗*p* < 0.01, ∗∗∗*p* < 0.001).

We designed two strategies to better understand how housing conditions may affect the health of female cynomolgus monkeys. In the first strategy, twenty-eight young female cynomolgus monkeys (2–3 years old) were housed singly for at least one month, then were randomly assigned into two groups: single to single (n = 15) and single to group (n = 13) (Fig. 1A). Monkeys included in the “single to single” group were housed alone in single cages throughout the whole experiment and served as single-caged control. The other monkeys assigned to the “single to group” treatment were housed together in one cage sufficiently large to accommodate all animals throughout the experiment. In the second strategy, twenty middle-aged female cynomolgus monkeys (10–15 years old) that lived in socially housed conditions for longer than one month were divided into two groups: group to group (n = 8) and group to single (n = 12) (Fig. 1B). Monkeys in “group to group” treatment were housed together in one large cage throughout the whole experiment and served as the grouped control, while monkeys in “group to single” treatment were separated into individual cages during the experiment. The time points for physical examination, blood and feces collection were illustrated in Fig. 1A and B, respectively.

First, we performed routine physical examinations of all monkeys in all treatment groups. In the first strategy, the body weights of monkeys in the two groups were comparable at the beginning of the experiment (day 0) (Fig. 1C). However, the percentage body weight gain of monkeys subjected to the “single to single” housing condition was significantly lower than that of “single to group”, especially from day 60 to day 210 (Fig. 1C). Accordingly, the body mass index (BMI) values of “single to group” monkeys were higher than that of single-caged controls (single to single) from day 90 to day 210 (Fig. 1C). Both the average heart rate (bpm) and body temperature (°C) of these two monkey groups varied significantly during the experiment (Fig. S1A). In the second strategy, the monkeys belonging to “group to single” treatment exhibited significant decreases in percentage body weight and BMI change when compared to the “group to group” controls from day 7 to day 120 (Fig. 1D). The heart rate (bpm) and body temperature (°C) of two monkey groups in the second strategy were stable over the experimental timecourse, with no significant differences observed between two groups (Fig. S1B). These results suggested that social deprivation can inhibit the weight gain of young monkeys and lead to a reduction on body weight in middle-aged monkeys.

**Fig. 2:**
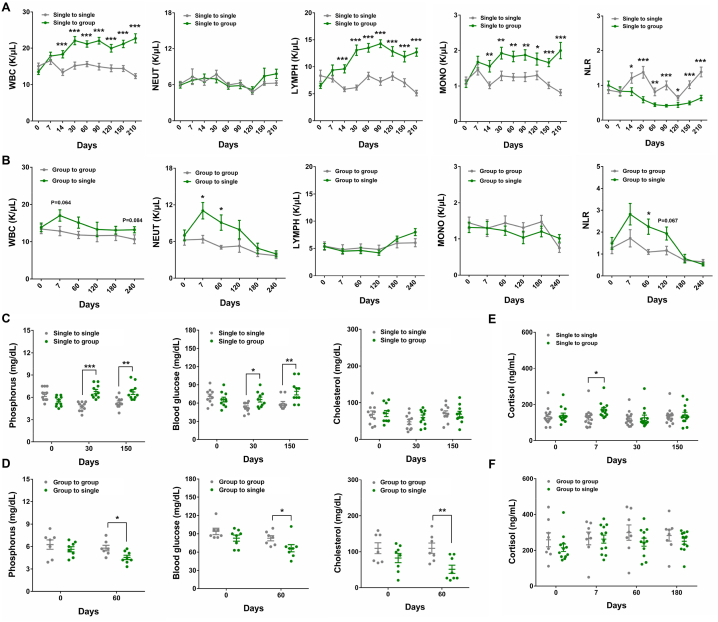
The routine blood and biochemical indexes of single-caged and grouped female cynomolgus monkeys (A) The comparison of routine blood indexes between the “single to single” (n = 15) and “single to group” (n = 13) young monkeys. (B) The comparison of routine blood indexes between the “group to group” (n = 8) and “group to single” (n = 12) middle-aged monkeys. (C) The comparison of serum phosphorus, glucose and cholesterol between the “single to single” (n = 10) and “single to group” (n = 10) young monkeys. (D) The comparison of serum phosphorus, glucose and cholesterol between the “group to group” (n = 7) and “group to single” (n = 8) middle-aged monkeys. (E) The serum concentration of cortisol in “single to single”(n = 15) and “single to group”(n = 13) young monkeys. (F) The serum concentration of cortisol in“group to group”(n = 8) and“group to single”(n = 12) middle-aged monkeys. Data are presented as mean ± SEM. The statistical significance between two monkey groups was analyzed using *t*-test (∗*p* < 0.05, ∗∗*p* < 0.01, ∗∗∗*p* < 0.001).

Next, the routine blood and biochemical indexes were measured to gain thorough insights into the physiological conditions of single-caged and socially-housed monkeys. In the first strategy, the white blood cell (WBC), lymphocyte (LYMPH), and monocyte (MONO) counts increased significantly from day 0 to day 30 in the “single to group” monkeys, then remained at this plateau from day 30 to day 210 (Fig. 2A). Specifically, the value of WBC, LYMPH, and MONO in the “single to group” monkeys were increased nearly 50 % as compared to that of the “single to single” monkeys during the period from day 30 to day 210. Accordingly, the ratio of LYMPH increased in the “single to group” monkeys, while the neutrophil (NEUT) ratio decreased (Fig. S2A). However, these routine blood indexes showed negligible change in the “single to single” monkeys during the experiment (Fig. 2A). In the second strategy, no significant differences were detected in LYMPH or MONO between the “group to single” and “group to group” monkeys throughout the whole experiment (Fig. 2B). Notably, both WBC and NEUT counts sharply increased in the “group to single” monkeys from day 0 to day 7, then both parameters exhibited a gradual, consistent decrease from day 7 to day 120 (Fig. 2B). At day 7, the value of WBC in “group to single” monkeys were increased nearly 25 % as compared to that of the “group to group” monkeys, while the value of NEUT in the “group to single” monkeys was two-fold higher than that of the “group to group” monkeys. Consistent with these data, the NEUT ratio was also increased in “group to single” monkeys, whereas LYMPH and MONO ratios decreased (Fig. S2B). Additionally, to assess the health state of monkeys, the neutrophil to lymphocyte ratio (NLR), an index of systemic inflammation [24], was calculated for all monkey groups. This analysis revealed a significant decrease of NLR in single-caged young monkeys following relocation to social housing conditions (Fig. 2A), while it increased in middle-aged monkeys that moved from group housing to single cages (Fig. 2B). These results, in conjunction with changes in body weight described above (Fig. 1C and D), indicated that the overall health of socially-housed monkeys was better than that of monkeys kept in single cages.

Subsequent biochemical examination of serum samples indicated that phosphorus and blood glucose were elevated significantly following the relocation of single-caged young monkeys to grouped housing (Fig. 2C). Specifically, the value of phosphorus in the “single to group” monkeys were increased nearly 40 % and 20 % as compared to that of the “single to single” monkeys at days 30 and 150. As for blood glucose, it was increased nearly 20 % and 30 % as compared to that of the “single to single” monkeys at days 30 and 150. By contrast, these two parameters were decreased upon separation of socially housed middle-aged monkeys into single cages (Fig. 2D). It is noteworthy that serum cholesterol levels also decreased when the grouped middle-aged monkeys were divided into single cages (Fig. 2D), whereas no significant differences were observed between the two young monkey groups in the first strategy (Fig. 2C).

Taking the close relationship between cortisol and stress into consideration [[25], [26], [27], [28]], we assayed the serum cortisol concentration in all treatment and control monkeys. In the short period (day 0 to day 7) after moving single-caged young monkeys to grouped housing conditions, the serum cortisol concentrations increased sharply (about 30 % increment), then rapidly declined to normal levels and stabilized from day 30 to day 150 (Fig. 2E). However, no obvious cortisol differences were observed between the “group to group” middle-aged monkeys and the “group to single” controls throughout the experiment (Fig. 2F). This implied that grouping could induce severe short-term stress to young female cynomolgus monkeys, while no significant changes in serum cortisol were seen in middle-aged monkeys.

All these results suggested that the health status of female cynomolgus monkeys were impaired by confining them to single cages.

### 2.2 Stress-related differences in gut microbiota between single-caged and socially housed female monkeys

In order to characterize the changes of gut microbial community that potentially induced by housing conditions, we performed high throughput 16S rRNA gene sequencing. The basic sequencing statistics (Raw_reads, Filtered_reads, Merged_reads, and Valid_reads) were listed in Table S1 and Table S2. Analyses of gut microbial richness (Chao1 and Observed species) and diversity (Shannon and Simpson) in young and middle-aged female cynomolgus monkeys indicated that the microbial diversity showed no significant differences between the monkeys housed alone and in groups (Fig. S3).

**Fig. 3:**
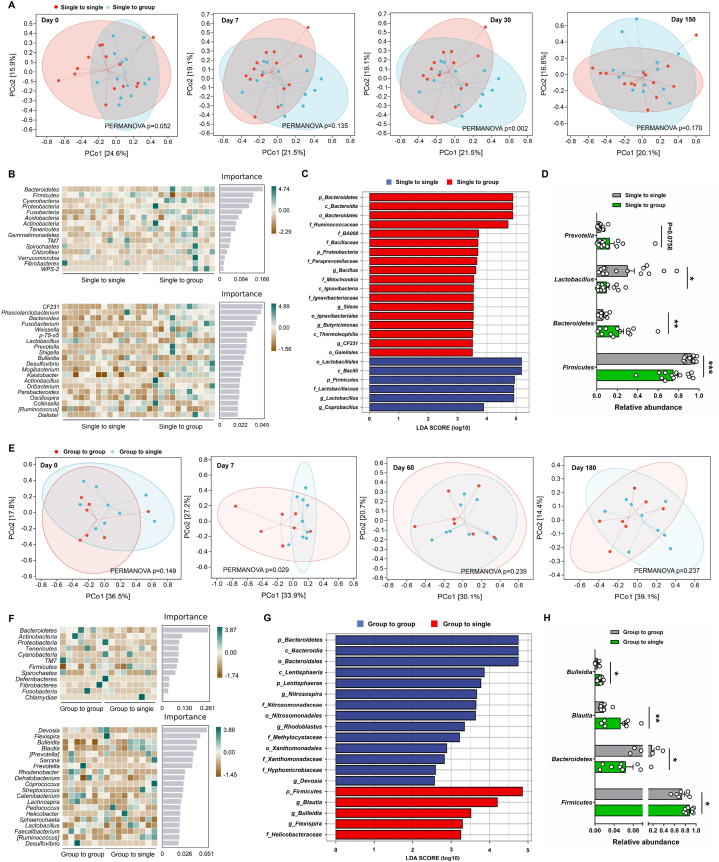
The gut microbiota composition of single-caged and grouped female cynomolgus monkeys (A) Principal coordinate analysis (PCoA) of weighted UniFrac distances between the “single to single” (n = 14) and “single to group” (n = 13) young monkeys at days 0, 7, 30 and 150. (B) Random forest-based identification of distinguishing taxa between the “single to single” and “single to group” young monkeys at day 30. The distinguishing taxa at phylum and genus level were listed. (C) LEfSe (Linear discriminant analysis Effect Size) analysis was used to analyze the gut microbial differences between the “single to single” and “single to group” monkey groups at day 30. LDA was set as 3.5. (D) Relative abundance of *Firmicutes*, *Bacteroidetes*, *Lactobacillus*, and *Prevotella* in the “single to single” and “single to group” monkey groups at day 30. (E) Principal coordinate analysis (PCoA) of weighted UniFrac distances between the “group to group” (n = 7) and “group to single” (n = 9) middle-aged monkeys at days 0, 7, 60, and 180. (F) Random forest-based identification of distinguishing taxa between the “group to group” and “group to single” middle-aged monkeys at day 7. The distinguishing taxa at phylum and genus level were listed. (G) LEfSe (Linear discriminant analysis Effect Size) analysis was used to analyze the gut microbial differences between the “group to group” and “group to single” monkey groups at day 7. LDA was set as 2.0. (H) Relative abundance of *Firmicutes*, *Bacteroidetes*, *Blautia*, and *Bulleidia* in the “group to group” and “group to single” monkey groups at day 7. Data are presented as mean ± SEM. The statistical significance between two monkey groups was analyzed using Mann-Whitney *U* test (Two-tailed) (∗*p* < 0.05, ∗∗*p* < 0.01, ∗∗∗*p* < 0.001).

We next compared the composition of gut microbiota between the two treatment groups in the first strategy. Principal coordinate analysis (PCoA) of a weighted UniFrac distance matrix revealed obvious gut microbial differences between the two monkey groups at day 30 (PERMANOVA, *p* = 0.002), while no gut microbiome differences were observed at days 0, 7, and 150 (Fig. 3A). The distinguishing taxa between these two monkey groups at day 30 were then identified by random forest and Linear discriminant analysis Effect Size (LEfSe) analyses (Fig. 3B and C). Specifically, Bacteroidia, Ruminococcaceae, Paraprevotallceae, *Bacillus*, and *Silene* were abundant in “single to group” monkey group; Firmicutes, *Lactobacillus*, and *Coprobacillus* were enriched in the “single to single” monkey group. At the phylum level, the relative abundance of Firmicutes was higher in “single to single” monkeys, while *Bacteroidetes* was lower at day 30 (Fig. 3D). At the genus level, the relative abundance of *Prevotella* was higher in the “single to group” monkeys, while the relative abundance of *Lactobacillus* was higher in “single to single” monkeys at day 30 (Fig. 3D). In the second strategy, PCoA analysis revealed significant gut microbial differences between the two monkey groups at day 7 (PERMANOVA, *p* = 0.029), while no gut microbiome differences were observed at days 0, 60, and 180 (Fig. 3E). The distinguishing taxa between these two monkey groups at day 7 were then identified by random forest and Linear discriminant analysis Effect Size (LEfSe) analyses (Fig. 3F and G). Specifically, Bacteroidetes, *Nitrosospira*, *Rhodoblastus*, and *Devosia* were abundant in “group to group” monkey group; Firmicutes, *Blautia*, *Bulleidia*, and *Flexispira* were enriched in the “group to single” monkey group. As for the differentially affected bacterial taxa, the relative abundance of *Firmicutes*, *Blautia*, and *Bulleidia* were higher in “group to single” monkeys, while the relative abundance of *Bacteroidetes* was lower at this time point (Fig. 3H).

**Fig. 4:**
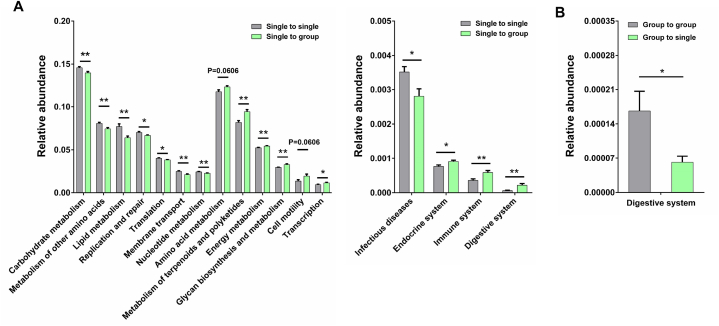
PICRUSt2-predicted pathway abundances in the gut microbiota of young and middle-aged female cynomolgus monkeys housed in isolation or social conditions (A) The predicted second-level KEGG pathways that significantly affected by relocating single-caged young monkeys (single to single) into a group cage (single to group) at day 30. (B) The predicted second-level KEGG pathway that significantly affected by separating socially-housed middle-aged monkeys (group to group) into single cages (group to single) at day 7. Data are presented as mean ± SEM. The statistical significance between two monkey groups was analyzed using Mann-Whitney *U* test (Two-tailed) (∗*p* < 0.05, ∗∗*p* < 0.01, ∗∗∗*p* < 0.001).

Then, PICRUSt2 was used to predicted the gut microbial function at specific time points in two strategies (day 30 in the first strategy and day 7 in the second strategy). In the first strategy, the KEGG pathways that related to amino acid metabolism, metabolism of terpenoids and polyketides, energy metabolism, glycan biosynthesis and metabolism, cell motility, transcription, endocrine system, immune system, and digestive system were enriched in the grouped young monkeys at day 30, while the functions related to carbohydrate metabolism, metabolism of other amino acids, lipid metabolism, replication and repair, translation, membrane transport, nucleotide metabolism, and infectious diseases were decreased (Fig. 4A). In accordance with the increased LYMPH and MONO in socially-housed young monkeys (Fig. 2A), the immune system of these grouped monkeys was improved when compared with the single-caged controls (Fig. 4A). Thus, the health state of grouped young monkeys was better that its single-caged controls. In the second strategy, although most of the KEGG pathways were kept unchanged in the separated middle-aged monkeys (group to single) at day 7 when compared with the grouped controls (group to group), the digestive system was significantly decreased (Fig. 4B). The decrease of digestive system in both the separated young and middle-aged monkeys (Fig. 4A and B) might be the key reason for their growth inhibition (Fig. 1C and D). All these results suggested that housing conditions could influence the health states of female cynomolgus monkeys by modulating their gut microbial composition and function.

We performed the linear regression analysis to investigate the correlations between the variation of gut microbes and the body weight, blood routine, and biochemical indexes. As shown in Fig. S4, the relative abundance of Firmicutes and *Lactobacillus* were negatively correlated with LYMPH, MONO, phosphorus, and blood glucose in the “single to single” and “single to group” monkeys at day 30, while the relative abundance of Bacteroidetes and *Prevotella* were positively correlated with LYMPH, phosphorus, and blood glucose. As for the “group to group” and “group to single” monkeys, the relative abundance of Firmicutes, *Blautia*, and *Bulleidia* were negatively correlated with the weight change and BMI change at day 7, whereas Bacteroidetes showed the opposite trend. Furthermore, the relative abundance of Firmicutes was positively correlated with NEUT, which was quite different from Bacteroidetes, *Blautia*, and *Bulleidia* (Fig. S5).

In summary, by comparing the influence of housing conditions (social housing and social isolation) on body weight, routine blood and biochemical parameters, and gut microbial composition and function in female cynomolgus monkeys (Table 1), we concluded that housing conditions can strongly affect the health status and gut microbiota of female cynomolgus monkeys.

**Table 1:** **Comparison of health status and gut microbial alternations of female cynomolgus monkeys housed in isolation (social isolation) or social conditions (social housing).** Red arrows () denote increased parameters in grouped young monkeys or separated middle-aged monkeys when compared with their corresponding controls, while green arrows () denote decreased parameters; the horizontal line (−) denotes the unchanged parameter.

	Social housing^a^	Social isolation^b^
**Routine physical indexes**		
Body weight (kg)		
BMI		
**Routine blood and biochemical indexes**		
WBC (K/μL)		
NEUT (K/μL)	–	
LYMPH (K/μL)		–
MONO (K/μL)		–
NLR		
Phosphorus (mg/dL)		
Blood glucose (mg/dL)		
Cholesterol (mg/dL)	–	
Cortisol (ng/mL)^c^		–
**Gut microbial composition and function**^d^		
*Firmicutes*		
*Bacteroidetes*		
Metabolism of terpenoids and polyketides		–
Endocrine system		–
Immune system		–
Digestive system		

a Data derived from the grouped young monkeys that relocated from single cages into a group cage in first strategy.

b Data derived from the single-caged middle-aged monkeys that separated from a group cage into single cages in the second strategy.

c The increased cortisol was derived from grouped young monkeys at day 7 in first strategy.

d Data derived from the grouped young monkeys at day 30 and single-caged middle-aged monkeys at day 7.

### 2.3 Improving health status of female cynomolgus monkeys by performing menstruation detection under socially housed condition

**Fig. 5:**
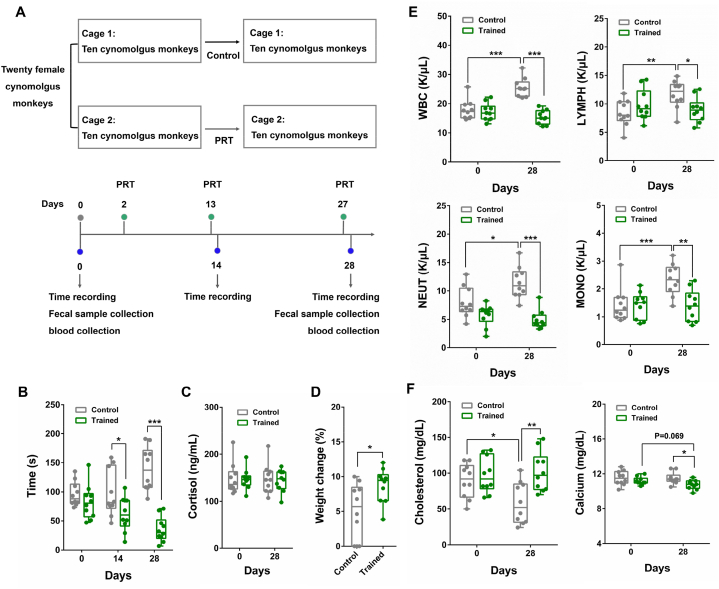
Facilitating menstruation detection through PRT and its effect on the health status of female cynomolgus monkeys (A) Schematic diagram showing the strategy for PRT. The monkeys in cage 1 were left untrained and used as control; the monkeys in cage 2 were trained for menstruation detection at days 2, 13, and 27. Time recording for menstruation detection was conducted at days 0, 14, and 28. The collection of feces and blood were performed at days 0 and 28. (B) The time used for menstruation detection between the trained (n = 10) and untrained (control, n = 10) monkeys at days 0, 14, and 28. (C) Comparing the serum cortisol concentration between the trained monkeys and its untrained controls. (D) Comparing the percentage change of body weight between trained (n = 10) and untrained control (n = 10) monkeys at day 28. The percentage body weight change was calculated based on the following fomula: (weight at day 28 minus weight at day 0)/weight at day 0. (E) Comparison of WBC, LYMPH, NEUT, and MONO between the trained (n = 10) and untrained control (n = 10) monkeys assayed before (day 0) and after (day 28) the training process. (F) Comparison of serum concentration of cholesterol and calcium between the trained (n = 10) and untrained control (n = 10) monkeys assayed before (day 0) and after (day 28) the training process. Data are presented as mean ± SEM. The statistical significance between two monkey groups was analyzed using *t*-test (∗*p* < 0.05, ∗∗*p* < 0.01, ∗∗∗*p* < 0.001).

Assisted reproductive technologies (ARTs) are fertility related treatment used to achieve pregnancy which involve the manipulation of both oocytes and sperm. As an essential step of ART in NHPs, menstruation is commonly monitored by isolating female monkeys in single cages [5]. As reported, positive reinforcement training (PRT), which teaches animal to voluntarily perform desired behaviors, can be applied to improve behavior and reactivity, thus reducing the stress-induced experimental confounds. For example, PRT had been used to enhance macaque cooperation during blood collection procedures, thus avoiding to confine the monkey in a squeeze cage [29]; Assisted with PRT, the macaque can insert their arms into a sleeve mechanism for venipuncture [30]. Thus, PRT acted as a useful strategy for enhancing the welfare of animals. Here, in light of the observed adverse effects on monkey health associated with single-cage housing (*e.g.*, reduced body weight or BMI and increased NLR) (Fig. 1, Fig. 2B), we next sought to determine whether PRT could be used to improve the health status of female cynomolgus monkeys by checking menstruation under social housing condition. To this end, twenty female cynomolgus monkeys (3–4 years old) that housed in two large cages (10 monkeys each) for longer than one month were used for detecting menstruation assisted with PRT. The monkeys that left untrained were set as controls (control group), while monkeys in the other cage were trained for menstruation detection (trained group) at days 2, 13, and 27 (Fig. 5A). The time required for detecting menstruation was recorded at days 0, 14, and 28. The fecal and blood samples were collected at days 0 and 28 (Fig. 5A). Compared with untrained monkeys, the time used for menstruation detection was significantly decreased in trained monkeys at day 14 (untrained, 97.4s; trained, 62.2s) and day 28 (untrained, 139.8s; trained, 35.3s) (Fig. 5B). Furthermore, the serum cortisol concentrations showed no significant difference between the trained and untrained monkeys (Fig. 5C), suggesting that the training process did not impact stress level in these female monkeys. In addition, the trained monkeys showed a higher percentage of body weight increase (8.7 %) than that of the untrained controls (4.9 %) from day 0 to day 28 (Fig. 5D), potentially due to trained monkeys showing greater cooperation when monitoring menstruation. Thus, PRT can be used to improve the health status of NHPs during scientific research.

We then assessed the physiological status of trained monkeys by performing routine blood test and blood biochemical assay. The results showed that WBC, LYMPH, NEUT, and MONO counts were kept unchanged in the trained monkeys between the samples collected before (day 0) and after the experiment (day 28) (Fig. 5E). By contrast, these parameters were all increased in the samples collected from untrained monkeys at day 28 (WBC, 43 %; LYMPH, 40 %; NEUT, 33 %; MONO, 65 %) (Fig. 5E). In addition, the NLR value showed no significant differences between the samples collected before and after the experiment in either the trained or untrained (control) groups (Fig. S6). Notably, the serum cholesterol concentration decreased significantly in the untrained controls at day 28 (35 %), while the concentration of calcium decreased slightly (3.8 %) in the trained monkeys compared with those parameters at day 0 (Fig. 5F). Both the phosphorus and blood glucose were kept unchanged between the trained monkeys and its untrained controls before and after the experiment (Fig. S7).

Since the health state of trained monkeys were better than its untrained controls (*e.g.*, higher percentage of weight increase in trained monkeys) (Fig. 5D), we concluded that PRT was an effective strategy for improving the welfare of laboratory NHPs during scientific research.

### 2.4 PRT exerts slight influence on gut microbiota of female cynomolgus monkeys

**Fig. 6:**
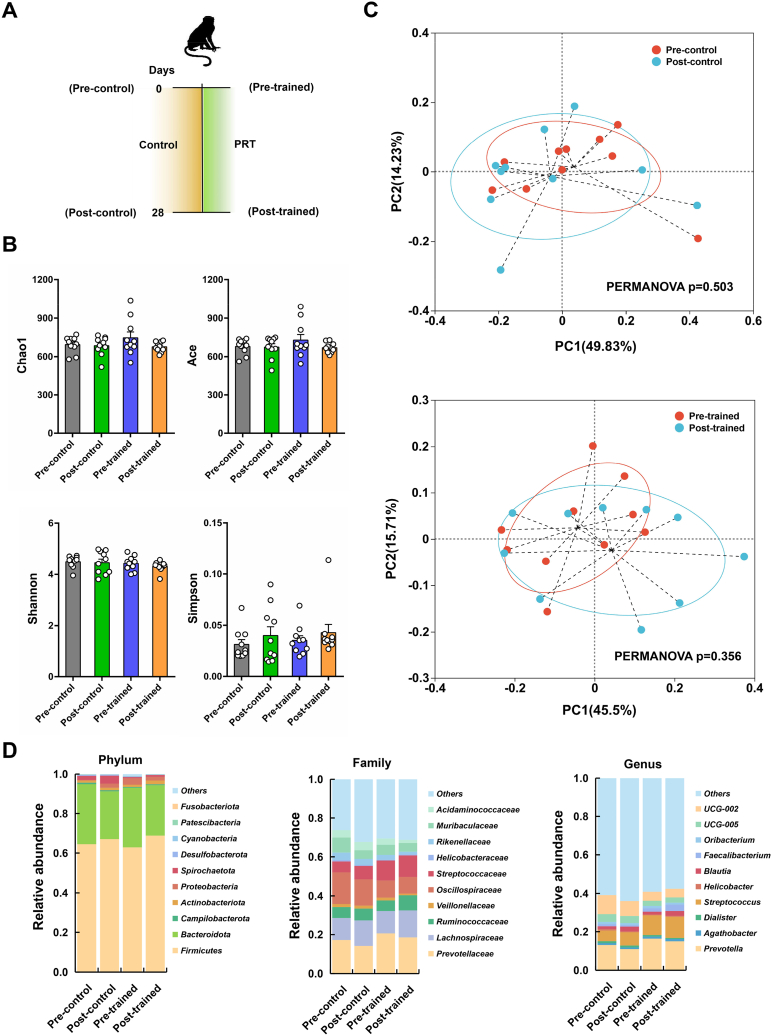
The comparison of gut microbiota between trained and untrained control monkeys (A) Schematic diagram showing the fecal samples collected for 16S rRNA gene sequencing. Pre-control, fecal samples collected from the untrained control monkeys before the experiment (day 0) (n = 10); Post-control, fecal samples collected from the untrained control monkeys after the experiment (day 28) (n = 10); Pre-trained, fecal samples collected from the trained monkeys before the experiment (day 0) (n = 10); Post-trained, fecal samples collected from the trained monkeys after the experiment (day 28) (n = 10). (B) The richness and sample diversity of gut microbiota between the trained and untrained control monkeys. (C) Principal coordinate analysis of weighted UniFrac distances between the gut microbiota of trained and untrained control monkeys collected before and after the experiment. (D) Relative abundance of top ten taxa assigned at phylum, family, and genus levels in trained and untrained control monkeys before and after the experiment.

To explore the effects of training process on the diversity and structure of gut microbiota in these monkeys, the fecal samples of trained and untrained monkeys that collected before (Pre-control and Pre-trained) and after (Post-control and Post-trained) the training experiment were used for 16S rRNA gene sequencing analysis (Fig. 6A). The basic sequencing statistics (Raw_reads, Filtered_reads, Merged_reads, and Valid_reads) were listed in Table S3. Venn diagram analysis showed that 824 OTUs were shared by all samples (*i.e.*, trained and untrained monkeys, before and after training) (Fig. S8A). No significant differences were found in the richness (Chao1 and Ace) and community diversity (Shannon and Simpson) of gut microbiota between the trained and untrained monkeys, suggesting that the training process did not affect gut microbiota diversity (Fig. 6B). PCoA of weighted UniFrac distances revealed that the gut microbiome of trained or untrained monkeys were quite similar before and after the training process (Fig. 6C). By analyzing the gut microbial differences between the trained and untrained monkeys at phylum, family, and genus levels, we found that the predominant taxa did not differ between pre- and post-experiment samples, except for a few differences in *Negativicutes*, *Megasphaera*, *Phoenicibacter*, and *Coprococcus* taxa (Fig. 6D and Fig. S8B). Inference of the predicted gut microbiota function by PICRUSt2 suggested that the top 20 second-level KEGG pathways showed no significant difference between the pre- and post-collected samples in trained and untrained monkeys (Fig. S8C). These results collectively implied that the training process exerts slight influence on gut microbiota composition and function of female cynomolgus monkeys.

## 3 Discussion

We examined the health status and gut microbiota of female cynomolgus monkeys housed in isolation or social conditions and identified the alterations associated with housing-related stress. Given the fact that social housing can benefit the health of female cynomolgus monkeys, we improved their welfare by checking menstruation under socially housed condition through positive reinforcement training (PRT).

As reported, chronic social separation can increase the afternoon (13:00–17:00) heart rates of female cynomolgus monkeys and exacerbate atherosclerosis [11]. However, this periodic increase of heart rate was not observed in our single-caged monkeys although the heart rate was assayed at around 14:00 (Fig. S1). Different from the monkeys that fed with normal diet in our study, the female monkeys with afternoon heart rate increase were fed with a cholesterol and fat containing diet. Then, diet and housing condition might jointly lead to the periodic increase of heart rate in female cynomolgus monkeys.

Since NEUT can respond to virtually all forms of inflammation and propagate itself [31], the increased NEUT in isolated middle-aged monkeys indicated that these monkeys might suffer from inflammation. This assumption needs to be further verified by assaying the changes of inflammatory factors in serum.

Phosphorus is an essential element that associated with many metabolic processes. Given the fact that chronic phosphorus deficiency can lead to growth retardation in children and skeletal abnormalities in adults [32], the lower phosphorus level in the single-caged young and middle-aged monkeys could be an important reason for their growth inhibition (Fig. 2C and D). Meanwhile, it needs to be further studied whether the decrease of phosphorus will bring additional adverse effects (*e.g.*, skeletal abnormalities) to the isolated middle-aged monkeys (Fig. 2D).

It has been reported that acute psychological stress can impair the cholesterol absorption of intestine and decrease serum cholesterol [33,34]. Consistent with this result, the serum cholesterol concentration was decreased in isolated middle-aged monkeys (Fig. 2D). However, it showed no differences between the grouped and single-caged young monkeys (Fig. 2C). The possible reason for this phenomenon might be caused by the different growth stage of young and middle-aged female monkeys. This hypothesis will be verified by studying the influence of aging on the stress-induced variations of routine biochemical indexes in our future work.

Although cortisol was closely related to the stress state of animals [[25], [26], [27], [28]], Koyama et al. reported that the changing of plasma cortisol showed sex differences under different housing conditions in juvenile cynomolgus monkeys and the concentration of plasma cortisol showed no significant difference between the female monkeys housed in isolation and in pair [7]. Consistent with this finding, no obvious changes in serum cortisol were observed between the grouped middle-aged female monkeys and its single-caged controls (Fig. 2F). Thus, the mechanism for sex-dependent variations of stress-induced parameters needs to be further verified and it will promote the consideration of sex difference in scientific research. Differently, when the single caged monkeys were grouped and lived in social housing condition, the cortisol levels increased in a short period and then returned to normal levels (Fig. 2E). This was quite in accordance with the previously reported results that the increased activity in social housing might stimulate higher cortisol levels [35]. Notably, it had been reported that moving the single-housed rhesus macaques to pair-housing and group-housing could reduce the rate of abnormal behaviors, while the serum cortisol level was less affected [10]. Similarly, when the rhesus macaques were experienced with quarantine-like procedures, the pair-housed macaques exhibited lower undesirable behaviors and less diarrhea than single-housed subjects, while no significant differences in cortisol levels were observed [36]. All these results indicated that the behavioral and physiological outcomes do not always cohere and then emphasized the importance of evaluating the behavioral outcomes of NHPs that suffered from the variation of housing conditions.

Deprivation of social interaction can reduce the richness and diversity of gut microbiota in rats [17]. However, in our study, this phenomenon was not observed in the monkeys housed in isolation condition (Fig. S3). This microbial difference between rats and monkeys might arise from the fact that rats were coprophagic, a habit that will facilitate the fecal-oral transmission of gut microbiota between animals housed together. So, the gut microbial richness and diversity was changed greatly in individually housed rats, but not for NHPs. As to the function of specific genera, the bacteria belonged to *Prevotella* had been well studied for its complex carbohydrate consumption ability and could benefit the host by promoting adsorption of nutrients [37]. Then, the decreased *Prevotella* in single-caged young monkeys (day 30) might be one possible reason for their growth inhibition (Fig. 1, Fig. 3D). *Lactobacillus* microbes can inhibit the growth of pathogenic organisms and were considered as protective organisms in intestine. However, in some cases, *Lactobacillus* had been viewed as pathogens in immunocompromised hosts [[37], [38], [39], [40]]. In our study, the immune status was suppressed in single-caged young monkeys as illustrated by decreased WBC, LYMPH, and MONO (Fig. 2A). Accordingly, the relative abundance of *Lactobacillus* was higher in these single-caged young monkeys at day 30 (Fig. 3D). Whether the increased relative abundance of *Lactobacillus* could bring adverse influences on female cynomolgus monkeys was an important issue that need to be further studied. Thus, verification of the function of these altered microbial taxa will help us to improve the health status of isolated monkeys by adjusting their gut microbiota composition.

Since non-human primates are prosocial animals and social deprivation could lead to stress that alters physiology, especially HPA axis dysregulation, social housing, either in pairs or groups was highly recommended by the guide for the care and use of laboratory animals (National Research Council, 2011) as well the animal welfare act. As reported, PRT, a useful method to bridge cooperation between trainers and subjects, had been used to teach animal to voluntarily perform desired behaviors during specific scientific experiment, thus reducing the stress-induced experimental confounds. To facilitate the process of PRT in research facilities, the experienced trainers should be familiar with the socially-housed animals firstly and then gained trust from animals by rewarding them when they performed the desired actions. Until now, PRT had been used in various NHPs and regarded as useful strategy for enhancing the welfare of experimental animals [29,30]. In our study, we found that housing in isolation can affect the health status and gut microbial composition and function of female cynomolgus monkeys. Notably, social housing with PRT rather than isolation for common protocols such as menstruation monitoring can preserve the normal physiological function and health of female monkeys. Besides, assisted with PRT, a series of scientific experiments including medicine injection, blood collection, and behavior monitoring could be performed by housing the NHPs in pairs or groups. All these results stressed the notion that PRT was an effective strategy for improving the welfare of laboratory NHPs during scientific research.

In summary, our results contribute to a more comprehensive understanding about the impact of housing conditions on the health and gut microbiome of female cynomolgus monkeys. Given that laboratory NHPs are commonly subjected to social deprivation in a series of scientific researches, the results derived from these stressed animals might mislead the researcher and affect the outcome of scientific or pre-clinical studies. Thus, training NHPs to cooperate with scientific procedures under socially housed condition will not only ensure their welfare but also enhance the accuracy and reliability of scientific results.

### 3.1 Limitations of the study

This study had some limitations. As reported, the responsiveness of juvenile monkeys to environmental changes differs between males and females [7]. In this work, constrained by the shortage of male cynomolgus monkeys, the effects of housing in groups or isolation on health and gut microbiota were studied in females, thus our conclusions should not be extended to males without further verification. Moreover, the variation in gut microbiota was determined by 16S rRNA gene sequencing. Thus, the stress-related changes in microbiota only reflect the genus level. Future work will use the metagenomics sequencing strategy to identify the specific bacteria that affected by housing conditions. Besides, although our study analyzed the correlations between the gut microbial alternations and the changes of weight, blood routine, and biochemical indicators, it is still curious whether the change of housing conditions leads to the changes in the physiological parameters firstly, which in turn leads to changes in gut microbiome or vice versa. Thus, more efforts should be done to deciphering the causal relationship between the gut microbiota and the host physiological parameters.

## CRediT authorship contribution statement

**Yunpeng Yang:** Writing – review & editing, Writing – original draft, Supervision, Project administration, Investigation, Formal analysis, Data curation, Conceptualization. **Yong Lu:** Resources. **Changshan Gao:** Resources. **Yanhong Nie:** Resources. **Hongfei Wang:** Resources. **Yufei Huang:** Project administration. **Haiyan Dong:** Resources. **Qiang Sun:** Writing – review & editing, Validation, Supervision, Project administration, Investigation, Funding acquisition, Formal analysis, Data curation, Conceptualization.

## Inclusion and diversity

We support inclusive, diverse, and equitable conduct of research.

## Ethics declaration

This study was reviewed and approved by the animal use and care committees of the CAS Center for Excellence in Brain Science and Intelligence Technology, Chinese Academy of Sciences, with the approval number: CEBSIT-2022016.

## Data availability statement

Raw data files for 16S sequencing available at BioProject database, ID number: SRP371903, SRP370609 and SRP371852.

## Declaration of competing interest

The authors declare that they have no known competing financial interests or personal relationships that could have appeared to influence the work reported in this paper.

## Figures and Tables

**Table:** 

REAGENT or RESOURCE	SOURCE	IDENTIFIER
**Critical commercial assays**		
Catalyst One® Chemistry Analyzer	Catalyst One	Cat# IDEXX
Human cortisol ELISA kit	BBI	Cat# D711340
OMEGA Soil DNA Kit	Omega Bio-Tek	Cat# M5635-02
Vazyme VAHTSTM DNA Clean Beads	Vazyme	Cat# N411-03
AxyPrep DNA Gel Extraction Kit	Axygen Biosciences	Cat# AP-GX-250
Quant-iT PicoGreen dsDNA Assay Kit	Invitrogen	Cat# P11496
NovaSeq 6000 SP Reagent Kit (500 cycles)	Illumina	Cat# 20029137
**Oligonucleotides**		
Forward primer for 16S rRNA gene sequencing	5′-ACTCCTACGGGAGGCAGCA-3′	N/A
Reverse primer for 16S rRNA gene sequencing	5′-GGACTACHVGGGTWTCTAAT-3′	N/A
**Software and algorithms**		
QIIME2	Bolyen et al., 2019; Caporaso et al., 2010	https://qiime2.org/
PICRUSt2	Langille et al., 2013	http://picrust.github.io/picrust/
R software	N/A	https://www.r-project.org/
Prism	N/A	https://www.graphpad.com/

## Resource availability

### Lead contact

Further information and requests for resources and reagents should be directed to and will be fulfilled by the lead contact, Qiang Sun (qsun@ion.ac.cn).

## Materials availability

This study did not generate new unique reagents.

## Method details

### Experimental cynomolgus monkeys

Cynomolgus monkeys were housed at Songjiang Non-human Primate Facility of Center for Excellence in Brain Science and Intelligence Technology, Chinese Academy of Sciences. The size of single cages used in this study were 0.7 m × 0.9 m × 1 m and the size of big cages were 2.15 m × 3 m × 10 m. At the end of our experiment, all the single-caged monkeys were grouped and housed in big cages.

In the training experiment, twenty female cynomolgus monkeys with age ranging from 3 to 4 years old and housed in two big cages (10 monkeys each) for at least one month were used. The monkeys in cage 1 (n = 10) were kept untrained and served as control, while monkeys in cage 2 (n = 10) were trained for menstruation detection at days 2, 13, and 27. The monkeys were trained once a day at day 2, 13, and 27 and the intervention time was set as 2 h. Menstruation detection was performed to all the monkeys in two groups at days 0, 14, and 28.

Cynomolgus monkeys were housed with controlled 12-h light/12-h dark schedule, controlled temperature (21–25 °C), humidity (∼50%–60 %) and continuous access to municipal water. These monkeys had no antibiotic exposure within 2 months during sample collection. All the monkeys were fed with nutritional granules twice a day (200 g per monkey at 8:00 a.m. and 3:00 p.m.) and vegetables and fruits (100 g per monkey at 10:00 a.m.) to provide essential nutrition and vitamins.

### Physical examination, routine blood examination and biochemical analysis

All the monkeys were carefully examined by experienced working staffs. The physical examination items include weight (kg), BMI, heart rate (BMP), and body temperature (°C). For the blood routine and biochemical analysis, the blood samples of female cynomolgus monkeys were collected at 7:00 a.m., the time point before feeding, and analyzed with chemistry analyzer (Catalyst One, IDEXX).

To evaluate the weight of monkey, the experienced working staff will fix the monkeys by putting their hands on the back. Then, both the staff and monkeys were weighted simultaneously and the weight was labeled as “staff + monkey”. Finally, the weight of monkeys was calculated by subtracting the weight of staff from the weight of “staff + monkey”. For blood draws, the monkeys were fixed as described above. Then, the working staff performed blood draws by putting the hands of the monkey out of the cage. To calculate the BMI, the height of the fixed monkeys was assayed by measuring the distance between the head and the bottom of tail. The heart rate of the fixed monkeys was assayed by using the portable digital heart rate stethoscope. The body temperature was assayed by measuring the anal temperature. These descriptions were added in the methods section in our revised manuscript.

### Enzyme-linked-immunosorbent assay (ELISA)

Serum was collected and used for enzyme-linked-immunosorbent assay (ELISA). The serum cortisol concentration was assayed by using human cortisol ELISA kit (Sangon Biotech, D711340) according to the protocol.

### Positive reinforcement training (PRT)

Monkeys used in this work are trained for menstruation detection by skillful staffs. During the training process, the trainer will show a hand and give explicit instructions (good/stop) to stop the monkey from moving and touch the back and tail gently for 2–3 min. All the trained monkeys will be rewarded with peanuts at the end of the training. Then, the trained monkeys will be subjected to menstruation detection the day after training and the time spent on this process is recorded. The training process will be stopped when all the monkeys can keep still after giving the good/stop instruction and be touched by the trainer.

### Fecal sample collection and 16S rRNA gene sequencing

Fresh fecal samples were collected directly into sterile tubes by experienced working staffs, frozen into liquid nitrogen immediately and then stored at −80 °C. Total genomic DNA samples were extracted using the OMEGA Soil DNA Kit (M5635-02) (Omega Bio-Tek, Norcross, GA, USA), following the manufacturer's instructions. The sample collected from the environment was used as negative control. During the PCR amplification, no-template-controls were also involved. The quantity and quality of genomes were measured with NanoDrop NC2000 spectrophotometer (Thermo Fisher Scientific, Waltham, MA, USA) and agarose gel electrophoresis, respectively. Then, the V3-V4 region of 16S rRNA was amplified using the primer pairs 338F (5′-ACTCCTACGGGAGGCAGCA-3′) and 806R (5′-GGACTACHVGGGTWTCTAAT-3′). The primers were labeled with sample-specific 7-bp barcodes for multiplex sequencing. After amplification, the PCR amplicons were purified with Vazyme VAHTSTM DNA Clean Beads (Vazyme, Nanjing, China) and quantified using the Quant-iT PicoGreen dsDNA Assay Kit (Invitrogen, Carlsbad, CA, USA). Equal amounts of amplicons were used for paired-end 2 × 250 bp sequencing using the Illlumina NovaSeq platform with NovaSeq 6000 SP Reagent Kit (500 cycles) at Shanghai Personal Biotechnology Co., Ltd (Shanghai, China). Raw sequence data were demultiplexed using the demux plugin following by primers cutting with cutadapt plugin. Sequences were then quality filtered, denoised, merged and chimera removed using DADA2 plugin [41].

### Data analysis

The sequences were analyzed using QIIME2 and R packages (v3.2.0). α-diversity was calculated using the OTU table in QIIME2; β-diversity was performed using Jaccard metrics to investigate the structural variation of microbial communities across samples. Principal coordinate analysis (PCoA) of weighted UniFrac distances between monkey groups was displayed by WGCNA package, stat packages and ggplot2 package in R software (Version 2.15.3). Based on the occurrence of OTUs across samples/groups regardless of their relative abundance, Venn diagram was generated to visualize the shared and unique OTUs among groups using R package “VennDiagram” [42]. Random forest analysis was applied to discriminating the samples from different groups using QIIME2 with default settings. Moreover, the statistically significant biomarkers were identified using linear discriminant analysis (LDA) effect size (LEfSe). Microbial functions were predicted by PICRUSt2 (Phylogenetic investigation of communities by reconstruction of unobserved states) upon KEGG (https://www.kegg.jp/) databases.

### Statistical analyses

The statistical significance of physical, routine blood and biochemical parameters between two monkey groups at different time points were analyzed using student *t*-test (unpaired, two-tailed); the gut microbial changes at phylum, family, and genus levels between two monkey groups were analyzed using student *t*-test (unpaired, two-tailed); the statistical significance corresponding to the predicted functional capabilities of microbial communities at second-level KEGG pathways between two monkey groups were analyzed using student *t*-test (unpaired, two-tailed). All statistical analyses were calculated using Graphpad software (version 7.0).
