# Supplementary material for: Effects of housing conditions on health and gut microbiome of female cynomolgus monkeys and improvement of welfare by checking menstruation under socially housed condition
Source: Heliyon. 2025 Jan 12;11(2):e41912. doi: 10.1016/j.heliyon.2025.e41912 (PMC11786677; doi:10.1016/j.heliyon.2025.e41912)
Supplement: Multimedia component 4 [file mmc4.docx]

**Supplementary information for:**

**Effects of housing conditions on health and gut microbiome of female cynomolgus monkeys and improvement of welfare by checking menstruation under socially housed condition**


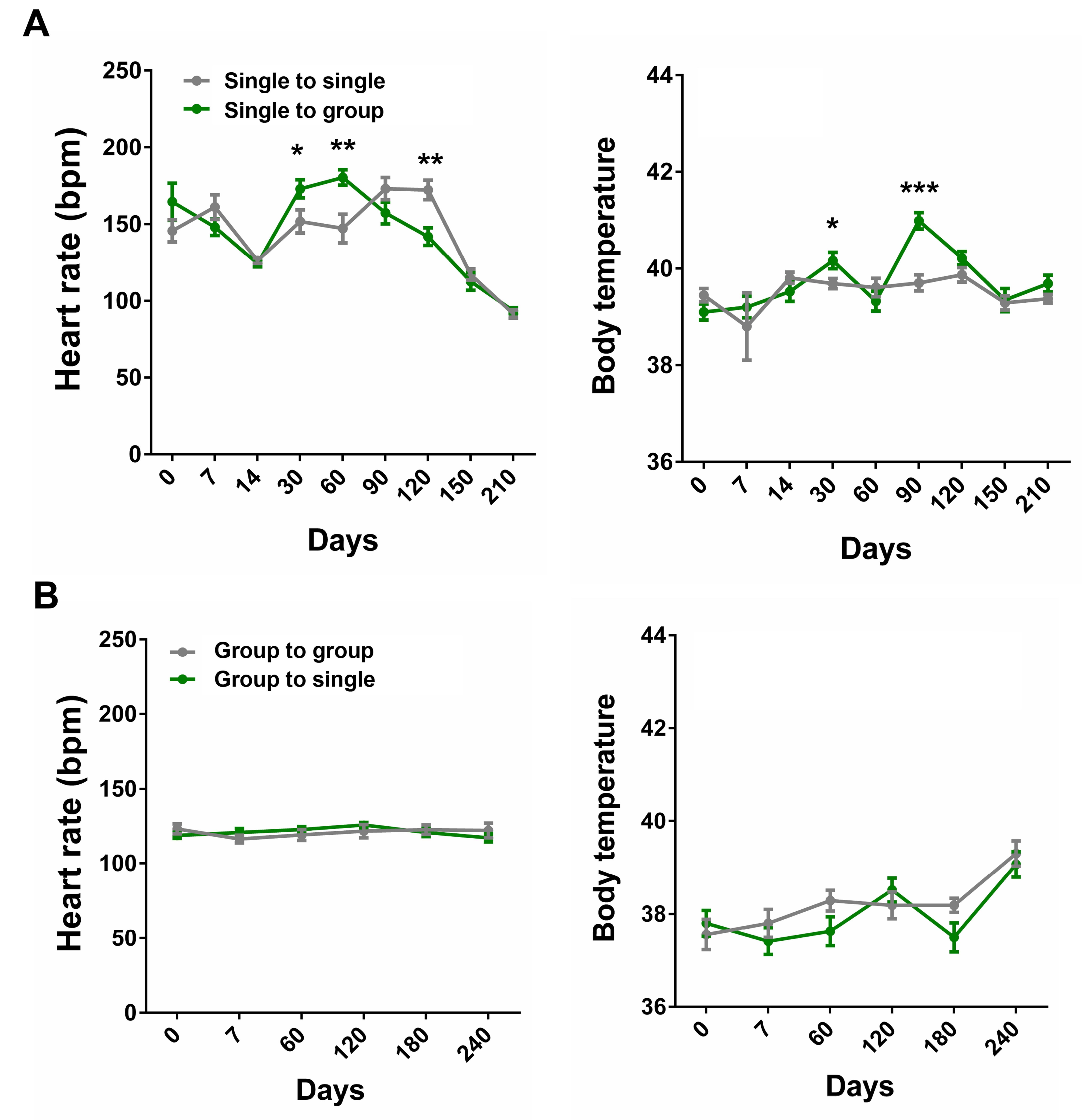


**Supplementary Figure 1. The physical status of female cynomolgus monkeys housed in isolation and social conditions**

(A) The heart rate (bmp) and body temperature (℃) between the grouped (single to group) and single-caged (single to single) young monkeys.

(B) The heart rate (bmp) and body temperature (℃) between the grouped (group to group) and single-caged (group to single) middle-aged monkeys.

Data are presented as mean±SEM. The statistical significance between two monkey groups was analyzed using t-test (**p*<0.05, ***p*<0.01, ****p*<0.001).


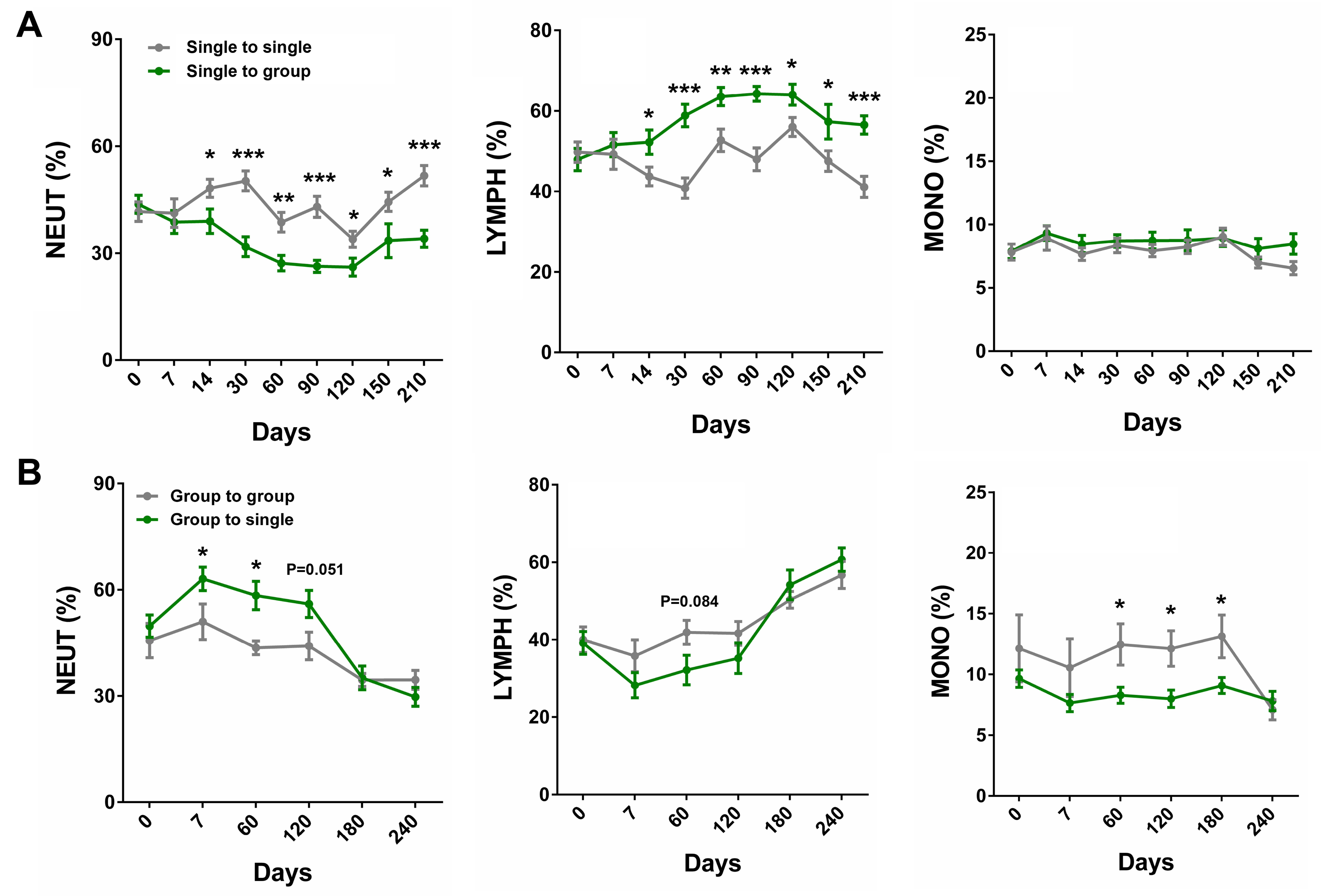


**Supplementary Figure 2. Ratio of routine blood indexes in isolated and socially housed monkeys**

(A) Comparing the ratio of NEUT, LYMPH, and MONO between the grouped (single to group) and single-caged (single to single) young monkeys.

(B) Comparing the ratio of NEUT, LYMPH, and MONO between the grouped (group to group) and single-caged (group to single) middle-aged monkeys.

Data are presented as mean±SEM. The statistical significance between two monkey groups was analyzed using t-test (**p*<0.05, ***p*-value<0.01, ****p*<0.001).





**Supplementary Figure 3. Gut microbial richness and diversity of female cynomolgus monkeys housed in isolation and social conditions**

(A) Comparison of gut microbial richness (Chao1 and Observed species) and diversity (Shannon and Simpson) between the grouped (single to group) and single-caged (single to single) young monkeys.

(B) Comparison of gut microbial richness (Chao1 and Observed species) and diversity (Shannon and Simpson) between the grouped (group to group) and single-caged (group to single) middle-aged monkeys.

Data are presented as mean±SEM.


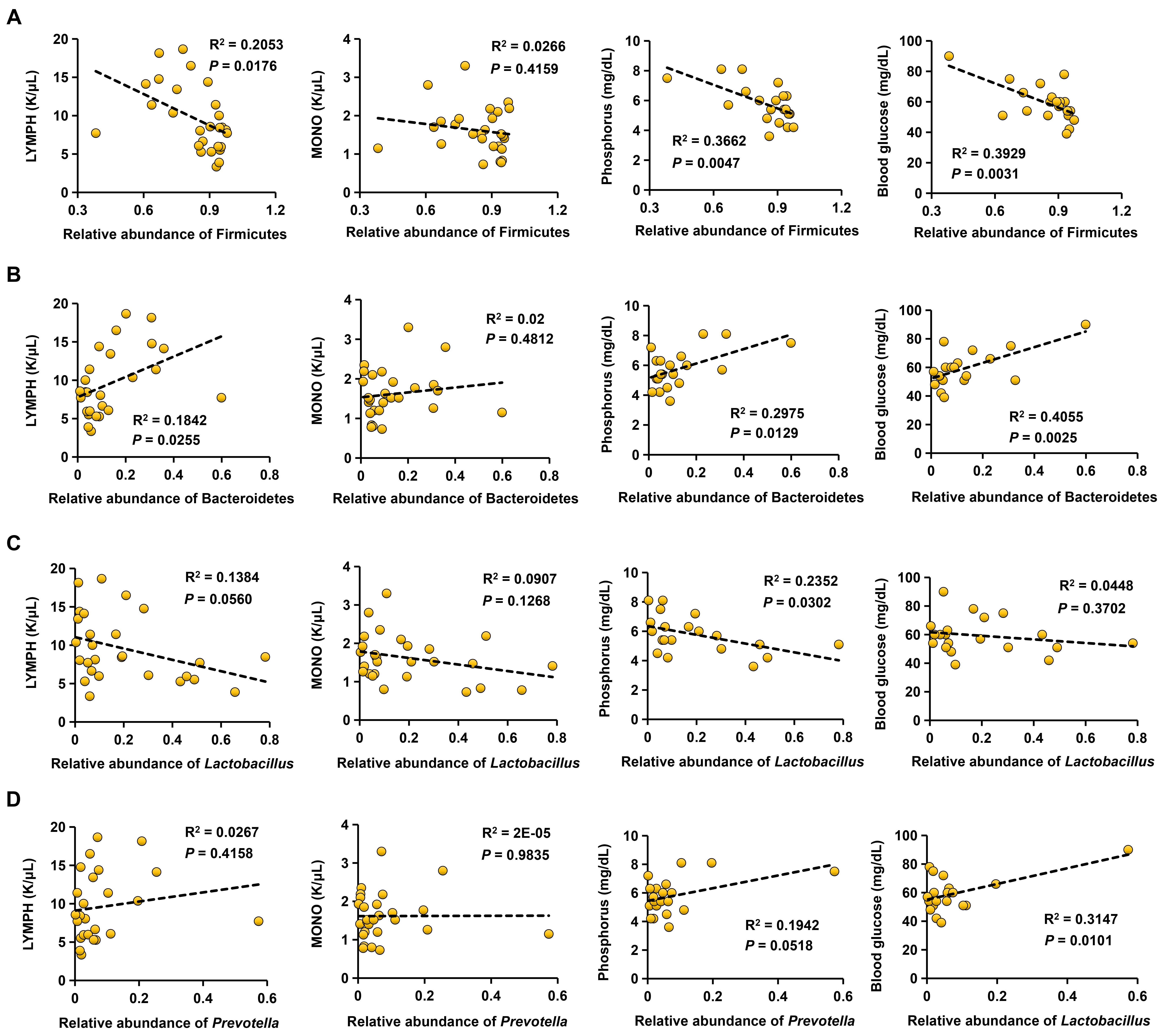


**Supplementary Figure 4. The correlations between the relative abundance of Firmicutes (A), Bacteroidetes (B), *Lactobacillus* (C), and *Prevotella* (D) and LYMPH, MONO, Phosphorus, and blood glucose in the “single to single” and “single to group” monkeys at day 30.**


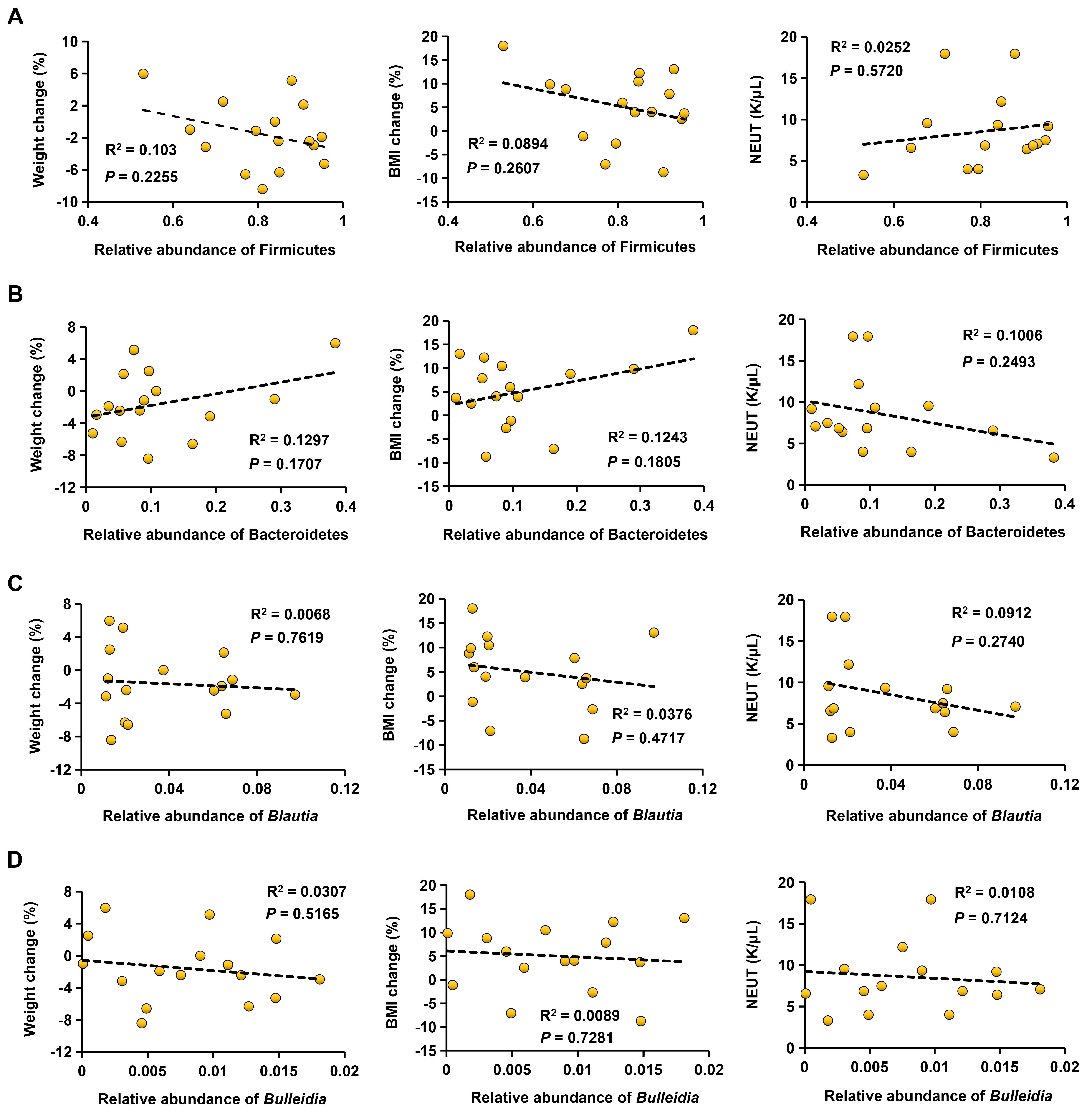


**Supplementary Figure 5. The correlations between the relative abundance of Firmicutes (A), Bacteroidetes (B), *Blautia* (C), and *Bulleidia* (D) and weight change, BMI change, and NEUT in the “group to group” and “group to single” monkeys at day 7.**


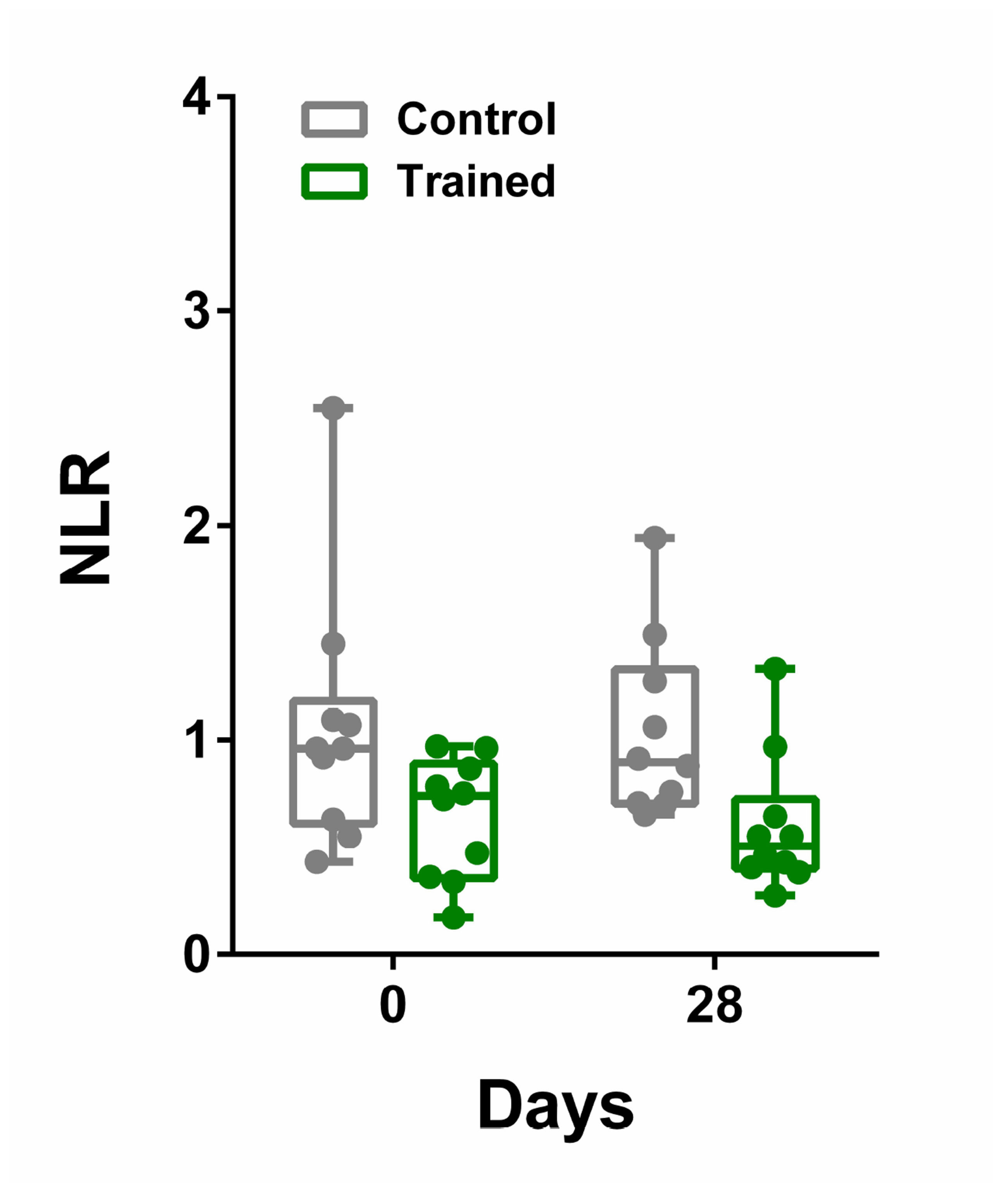


**Supplementary Figure 6. The neutrophil to lymphocyte ratio (NLR) of trained and control monkeys assayed before (day 0) and after (day 28) the training process.** Data are presented as mean±SEM.


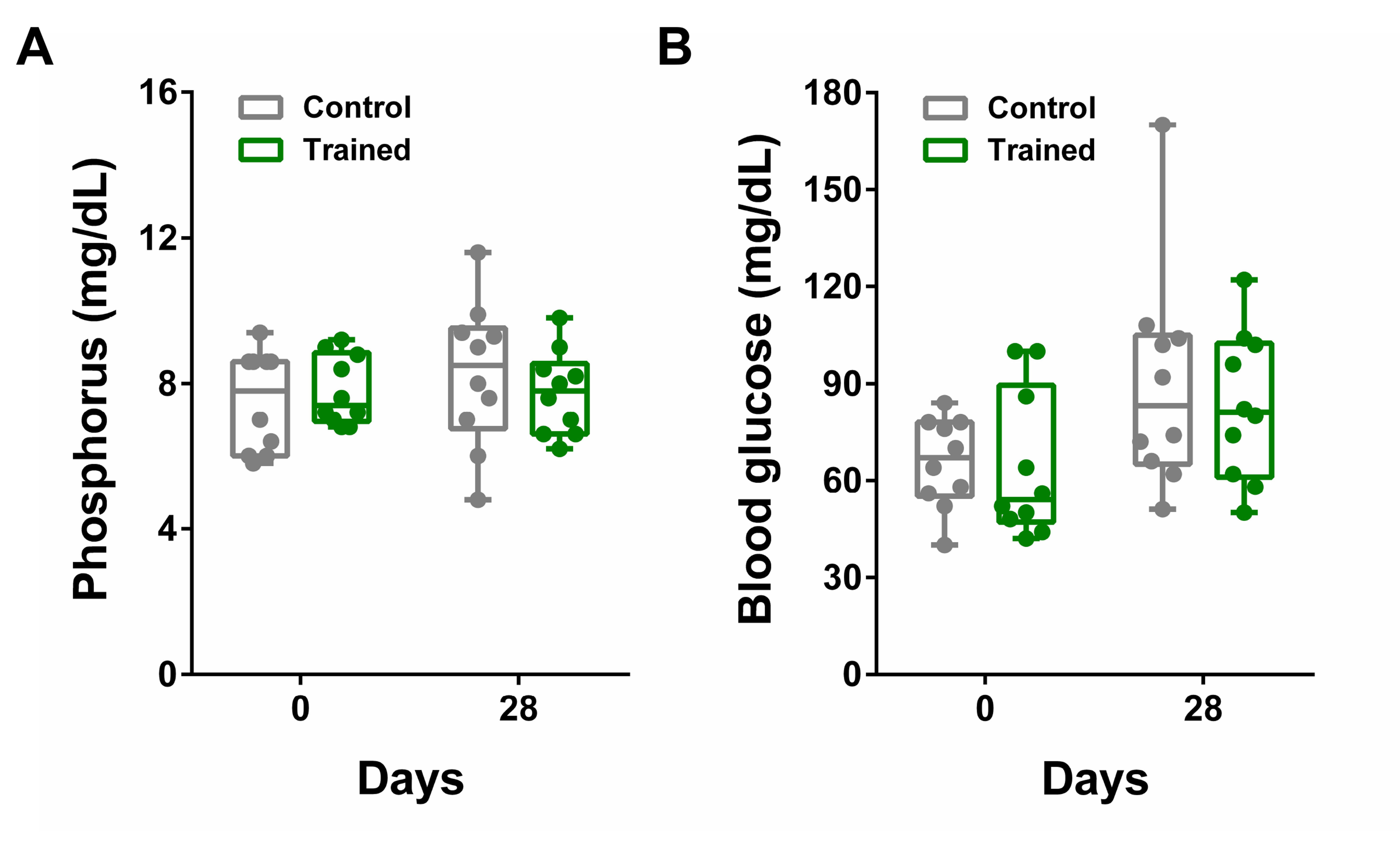


**Supplementary Figure 7. Comparison of serum concentration of phosphorus and glucose between trained (trained) and untrained (control) female cynomolgus monkeys**

(A) Comparing the serum phosphorus concentration between the trained and control monkeys assayed before (day 0) and after (day 28) the training process.

(B) Comparing the serum glucose concentration between the trained and control monkeys assayed before (day 0) and after (day 28) the training process.

Data are presented as mean±SEM.





**Supplementary Figure 8. Comparison of gut microbial composition and function between trained and untrained (control) female cynomolgus monkeys**

(A) Venn diagram illustrating the number of OTUs identified in control and trained monkeys before and after the experiment.

(B) Relative abundance of *Negativicutes*, *Megasphaera*, *Phoenicibacter*, and *Coprococcus* in trained and control monkeys before and after the experiment. Data are presented as mean±SEM. The statistical significance between Pre-control/trained or Post-control/trained monkey groups was analyzed using t-test (**p*<0.05, ***p*<0.01).

(C) Comparing the gut microbial function of trained and control monkeys before and after the experiment. The function of gut microbial communities was analyzed with PICRUSt2 and top 20 second-level KEGG pathways were listed. The statistical significance between Pre-control/trained and Post-control/trained monkey groups was analyzed using t-test and listed at right.
